# “AMR Dialogues”: a public engagement initiative to shape policies and solutions on antimicrobial resistance (AMR) in Thailand

**DOI:** 10.12688/wellcomeopenres.17066.2

**Published:** 2022-04-07

**Authors:** Tassawan Poomchaichote, Anne Osterrieder, Ravikanya Prapharsavat, Bhensri Naemiratch, Supanat Ruangkajorn, Chaiwat Thirapantu, Karnjariya Sukrung, Niyada Kiatying-Angsulee, Nithima Sumpradit, Sirima Punnin, Direk Limmathurotsakul, Phaik Yeong Cheah

**Affiliations:** 1 Mahidol Oxford Tropical Medicine Research Unit, Faculty of Tropical Medicine, Mahidol University, Bangkok, Thailand; 2 Centre for Tropical Medicine & Global Health, Nuffield Department of Medicine, University of Oxford, Oxford, UK; 3 Civicnet Foundation, Bangkok, Thailand; 4 Drug System Monitoring and Development Centre, Faculty of Pharmaceutical Sciences, Chulalongkorn University, Bangkok, Thailand; 5 Food and Drug Administration, Ministry of Public Health, Nonthaburi, Thailand; 6 Department of Tropical Hygiene, Faculty of Tropical Medicine, Mahidol University, Bangkok, Thailand

**Keywords:** antimicrobial resistance, Thailand, antibiotics, responsive dialogues, public engagement

## Abstract

**Background:** The use of antimicrobials in Thailand has been reported as one of the highest in the world in both the human and animal sectors. The objectives of this project are: (1) to improve understanding of the issue of antimicrobial resistance (AMR) among adult Thai communities and (2) to drive change through the national AMR policy to include context-specific and locally-driven solutions.

**Methods:** The project contains two components conducted in parallel: the “AMR Dialogues” public engagement project and the embedded evaluation of the project. We will bring together AMR stakeholders and members of the public through a series of conversation events to co-create an AMR stakeholder map, engagement strategy, and context-specific solutions to reduce the burden of AMR. There will be a combination of regional in-person events (‘regional conversations’) and national online events (‘national conversations’) with members of the public and AMR stakeholders. The conversations will follow this sequence: introduce and explore issues related to AMR, brainstorm solutions and finally propose promising/feasible solutions to take forward. Evaluation of the project will be conducted to assess if the AMR Dialogues objectives have been achieved using feedback forms and qualitative methods.

**Ethics:** Approval of the evaluation component of the project has been obtained from the ethics committee of the Thailand Institute for the Development of Human Subject Protection (IHRP2021059) and the Oxford University Tropical Research Ethics Committee (OxTREC 529-21).

**Dissemination:** The results of these conversation events will inform the next Thailand National Strategic Plan on AMR. The learning and outcomes will be disseminated to AMR policy makers, academic audiences, and participants of all the conversation events.

**Thaiclinicaltrials.org registration:** TCTR20210528003 (28/05/2021)

## Introduction

Antimicrobial resistance (AMR) is the ability of microorganisms, including bacteria, viruses, fungi, and parasites, to stop antimicrobial drugs (such as antibiotics, antivirals, antifungal drugs and antiparasitic drugs) from working against them ^1^. It has been estimated that globally there will be around 700,000 deaths each year caused by AMR bacterial infections ^2^. The human and economic cost of AMR is estimated to account for 10 million deaths a year and economic losses of US$100 trillion globally by 2050 ^2^. It is projected that a disproportionate number of AMR-related deaths are likely to occur in Asia because of the high burden of infectious diseases, poor monitoring and regulation of antimicrobials, high population density and growth, and antibiotics are sold widely over-the-counter often without a prescription ^2– 4^. A recent study estimated that there were over 97,000 deaths attributable to AMR in Southeast Asia alone ^5^. South-East Asia has been characterised as a region ‘at high risk of the emergence and spread of antibiotic resistance in humans’ ^6, 7^. In Thailand, there were an estimated 19,122 deaths in 2010 attributable to multidrug resistant hospital-acquired infections ^8^.

Thailand’s healthcare system is made up of a combination of a network of public health facilities and private healthcare providers and overseen by the Ministry of Public Health. Universal health coverage has been in place since 2002. In rural and poorer areas, primary healthcare is primarily provided by the public healthcare system, whereas in urban and more affluent areas, private providers play a larger role ^9^. The Thai Drug Act classes the majority of antibiotics as dangerous drugs which need to be dispensed by a pharmacist but do not require a prescription. Some antimicrobials are listed prescription drugs. Nevertheless, some antibiotics are sold illegally at informal stores. Antibiotic dispensing is not currently regulated and antibiotics can often be found in village stores and other shops without a pharmacist ^10^.

The Thai general public have a limited understanding of AMR ^11^, which is partly attributable to the challenge of communicating technical concepts of AMR in Thai language. Antibiotics are often confused with anti-inflammatory drugs in Thailand ^12^, that has led to a misunderstanding that antibiotics can treat muscle pain and inflammation ^12^. Antibiotics have become a quick fix for care, productivity, hygiene and inequity ^13^. Thailand has one of the highest antibiotic use in both human and animal sectors among countries that have published official data on national antibiotic consumption ^14, 15^. In 2017, Thailand used a total of 8,757 tons of antimicrobials ^16^, and in 2018, 10,133 tons ^17^.

For decades, Thailand had taken actions to address AMR such as establishing the National AMR Surveillance Center, Thailand (NARST) in 1997, and launching the Antibiotic Smart Use programme focusing on changing prescribing practices made by healthcare providers in 2007 ^18^.

In 2016, the Thailand National Strategic Plan on AMR (NSP- AMR), a 5-year plan from 2017 to 2021, was endorsed by the Cabinet as the first national plan to address the problem of AMR in Thailand ^19– 22^. The NSP-AMR comprises of six strategies. The “AMR Dialogues” project will integrate with the NSP-AMR’s Strategy 5 (public knowledge and awareness of appropriate use of antimicrobials) and if appropriate, provide input into Strategy 6 (governance mechanisms to develop and sustain AMR-related actions). It will inform the NSP-AMR process beyond 2021.

The Thailand “AMR Dialogues” project uses the Wellcome Trust’s toolkit “Responsive Dialogues (RDs) on Drug Resistant Infections” ^23^, which suggests a format for holding these Dialogues (see Methods). This projects builds on our previous engagement work around AMR such as “AMR Dictionary” ^1^, “antibiotic footprint” ^14^, and art and theatre projects around AMR in Thailand and Southeast Asia ^24– 26^, and complements existing campaigns such as the annual Antimicrobial Awareness Day/Week held since 2013 ^21^.

Previous and existing AMR campaigns have primarily focused on raising awareness through education and events ^22^. The current project focusses on engagement through a series of dialogues with different members of the community to generate solutions that are grounded in local realities and embrace ideas and views from the public. The project will bring together AMR stakeholders and members of the public to co-create an AMR stakeholder map, engagement strategy, and context-specific solutions with the aim of reducing the burden of AMR in Thailand.

The main objectives of this project are:

(1) to improve understanding of the issue of AMR among adult Thai communities and

(2) to provide a catalyst for improving the current national AMR policy, specifically Strategy 5 (public knowledge and awareness of appropriate use of antimicrobials) and to drive change through the national AMR policy to include context-specific and locally-driven solutions.

The intended long-term impacts of the project include empowerment of communities through co-creation of locally relevant solutions to AMR; and improved policies for reducing the burden of AMR across Thailand.

## Protocol

This protocol has been registered at Thaiclinicaltrials.org on the 28 ^th^ May 2021 (TCTR20210528003).

### Part 1: AMR Dialogues

There will be three phases in the project involving a series of dialogues or “conversations” held over 24 months.

All conversations will be led by members of the project team who have had experience and training in public engagement and facilitation, and trained using Wellcome Trust’s “Responsive Dialogues” toolkit ^23^. MORU has dedicated public engagement team (led by author PYC) and an extensive public engagement programme that involves facilitating traditional engagement activities as well as more innovative approaches such as science theatre initiatives ^14, 24, 26– 28^. Civicnet Foundation has experience facilitating workshops in communities and inspiring change. Where appropriate, we will ask participants to lead the breakout sessions. All sessions will be audio-recorded to facilitated note taking.

In all conversations, the project team will be cognizant of potential power dynamics due to age, social hierarchy and gender. 

***Phase I: Two or three planning conversations.*** The planning conversations are events comprising one or two-day meetings and held in Bangkok, Thailand at a conference venue led by members of the project team (PYC and NKA, female; CT, male). Approximately 20–25 attendees of all genders will participate in the conversations. From our experience facilitating workshops, 20–25 people are ideal to ensure that the sessions are small enough to be interactive and large enough to have a wide range of views ^27^. With this number we can also have breakout groups. The attendees will include AMR national policy makers, AMR researchers and experts, healthcare providers and non-governmental organization (NGO) representatives. The same participants will attend both meetings. Diverse stakeholders will be invited through professional networks, many of whom would have previously worked with project team members in AMR related or other healthcare projects.

The aim of the planning conversations is to understand the AMR ecosystem in Thailand, narrow down AMR focus, design the structure, and identify participants for the community conversations.

***Phase II: Seven community conversations.*** There are two types of community conversations: regional conversations and national conversations that will be conducted in parallel. By holding these two types of events, we hope to be as inclusive as possible and maximise the chance of having a diverse audience. 

Regional conversations will be led by CT (male) and TP (female) and national conversations will be led by TP (female), RP (female) and SR (male).

Participant selection criteria for community conversations:

1. Participants who have the capacity and means to drive and engage people in the community such as youth leaders. This will be a subjective assessment based on participants involvement in other AMR or health-related projects
2. Participants who have the commitment and are influential in the community such as religious leaders, teachers, village elders
3. Participants who could have an impact on behaviour change in antimicrobials use such as local healthcare workers e.g. nurses, pharmacists, doctors, village health volunteers
4. Participants who are familiar with the local context, lifestyles and culture of each region e.g. in the Northeastern region, we will invite people who live in slums, in the Central will invite people from labour camps, dormitory or migrant workers
5. Participants who have leadership characteristics such as community leaders. This assessment will be based on their involvement in community groups and committees.
6. Participants who are potential “solution experts” e.g. those who work in mass media, media influencers, science communication specialists, radio broadcasters, reporters
7. Participants who are longterm or frequent users of antimicrobials e.g. for treatment of tuberculosis, acne
8. Participants who are users of antimicrobials in the animal sector such as farmers
9. Participants from minority groups e.g. LGBTQ (lesbian, gay, bisexual, transgender and queer), disabled, hilltribes
10. Participants from different age groups, social groups, and educational levels
11. Participants with different occupations
12. Formal and informal prescribers/providers of antimicrobials

The regional conversations focus on regional issues and will be attended in person by local residents, while the online national conversations focus on national issues and will be attended by people from all over the country. We hope that with a combination of these two types of conversations, we will include voices of all of the above participant groups.

Regional conversations will be in-person three- or four-day sessions and held in four regions in Thailand (north, northeast, south and central) at conference venues/community halls. Approximately 20–25 attendees will attend the conversations from each region (total of 80–100). From our experience facilitating workshops, 20–25 people are ideal to ensure that the sessions are small enough to be interactive and large enough to have several breakout groups in-person) and a wide range of views ^27^. Each regional conversation will follow the following sequence as detailed in the Wellcome Trust’s ‘Responsive Dialogues’ toolkit: introduce and explore issues related to AMR in the region, brainstorm local solutions and finally choose promising/feasible solutions to take forward ^23^.

National conversations will consist of three online conversations and held three hours once a month for three months consecutively. Approximately 20–25 attendees will participate in the conversations. The same participants will be asked to attend all three sessions. Similar to the regional conversations, the online national conversations will follow the following sequence as detailed in the Wellcome Trust’s ‘Responsive Dialogues’ toolkit ^23^. The first session will introduce and explore issues related to AMR in the region, the second session will involve brainstorming locals solutions and the third session with involve participants proposing promising/feasible solutions to take forward. We will also plan to have virtual breakout groups.

***Phase III: Two final conversations.*** The events will be one or two-day meetings and held in Bangkok, Thailand at a conference venue. Approximately 20–25 attendees will participate in the conversations. The attendees will include AMR national policy makers, AMR researchers and experts, healthcare providers, non-governmental organizations (NGO), and communication and “solution experts”.

In these conversations, findings from the community conversations will be fed back to AMR stakeholders. Depending on what solutions have been proposed during the community conversations, we will invite the relevant “solution experts”. For example, if a social media campaign has been suggested targeting young people, we will invite social media experts and those with experience communicating with young people.

Participants for all the conversations will be invited in writing by the project team with details of the project. Participants will be asked to reply in writing or by phone should they accept the invitation. 

### Part 2: Evaluation

Evaluation will be conducted in parallel with all ‘AMR Dialogues’ events (planning conversations, regional conversations, national conversations, final conversations) to assess if the AMR dialogues objectives have been achieved (led by BN).

***Data collection strategies for evaluation and sample size.*** Feedback forms will be used at the end of each conversation event. Participants will be asked to complete a short feedback either on paper or online.

Daily reflections and feedback in writing will be used for events that run over multiple days (i.e regional conversations). An informal dialogue session will be set up at the end of each day which will take a maximum of 30 minutes. Participants will share their active observations and feedback on key issues. The daily reflection would help the event facilitators to adjust the conversation process better the next day. Participants are requested to write their reflections on a set of questions which aim to assess 1) their understanding or increased knowledge on AMR, 2) their level of awareness of AMR, and 3) their actions or solutions they think they would and could do regarding AMR. The writing activity will take maximum 30 minutes.

Focus group discussions (FGDs) will be used for events that run over multiple days (i.e. regional conversations). On the last day of event, participants who express their interest in joining the evaluation will be invited to share their active observations and feedbacks on key issues. We expect to run four FGDs of between 6–12 participants per FGD. This will take approximately 30 minutes to 1 hour per session.

Within a month after each event of the regional conversations, the last online national conversation, and the final conversations, people who express their interest in the in-depth interview will be invited to participate in the individual in-depth interviews. The interviews will take approximately 30 minutes to 1 hour each.

We anticipate that approximately 70 – 85 participants, including focus group discussions and in-depth interviews to participate in the evaluation.

FGDs and in-depth interviews will be conducted using a topic guide, led by AO and BN, both of whom are experts in evaluation and are not directly involved in facilitating the conversation events. The topic guides for focus group discussions and in-depth interviews, consent form and participant information sheet can be found as extended data ^29^.

***Participants and recruitment.*** The evaluation participants will be the attendees of any of the project’s conversation events. Participants who attend any of the AMR Dialogues public engagement events will be invited to participate in the AMR Dialogues evaluation project. As the evaluation process is embedded in the AMR Dialogues public engagement events, participants will be asked to indicate in the registration form when attending the conversation events, whether they will allow us to record and collect information of their daily reflection, feedback in writing, group discussion and follow-up in-depth interview. Information from any participants who do not wish to participate will not be collected. 

***Informed consent.*** Participants for all conversations will be invited in writing with all relevant details. If they choose to participate in the conversations (either in person or online), they will attend the conversations.

Participants who indicated their interest for the evaluation at time of registration will receive the participant information sheet. They will be allowed as much time as they wish to consider the information, the opportunity to question the project team and to decide whether they will be willing to allow their information to be collected and recorded. At this point, they will have the opportunity to decline. If they agree to participate, they will be asked to sign and date the latest approved version of the informed consent form before any study specific procedures are performed. Depending on the circumstances (coronavirus disease 2019 (COVID-19) restrictions and geographical location/internet access), this will be done either on paper or electronically.

Each participant has the right to withdraw from the conversation events or evaluation sessions at any time. Withdrawal of consent to participate will result in exclusion of the data for that participant from analysis and withdrawn participants will not be replaced. Participants are not required to give any reason for withdrawal. 

The audio recording and written data will be deleted if the participant decides to withdraw during the conversation events or the evaluation interviews. For those participating in the group discussion those portions of the audio, video recordings and written data that capture his/her views will be deleted.

***Data analysis.*** For conversation events, meeting minutes will be taken during all conversations. The project team will reflect and refine a set of solutions and ideas that will be emerged and will inform the responsive dialogues process.

For evaluation, the qualitative data will be translated where needed, transcribed, cleaned and exported into the latest version of NVivo (© QSR International Pty Ltd), the software that will be used to manage the data. Codes will be established for each participant to enable appropriate collation of data sets and sub-themes and themes for qualitative data.

Qualitative data analysis will be based on thematic content analysis, particularly Framework Analysis, given the policy implications of the planned work ^30, 31^, We will develop initial themes and categories through successive coding of the transcripts, and informed by the our project aims as well as issues derived from the literature. For the purpose of quality control , two researchers will independently develop their own coding tree. Frequent meetings will be held with the wider research team to discuss the findings.

***Data handling and record keeping.*** Direct access to the data will be granted to authorised representatives from the sponsor, ethics committees, and regulatory authorities to ensure compliance with regulations. Qualitative data includes audio recordings of focus group discussion and interviews, transcripts, written feedback, meeting minutes and field notes. Audio files will be transcribed verbatim and translated to English where necessary. Detailed summary notes will be made directly following the interview and during the focus group discussion with selected verbatim quotes being used. These audio files will be kept until they have been transcribed and the transcribed interviews will be kept securely. All audio files will be destroyed when all the transcripts have been completed and verified. 

De-identified data will be stored digitally and indefinitely to comply with the Wellcome Trust data sharing policies.

### Ethics, quality control and quality assurance procedures

Approval of the evaluation component of the project has been obtained from the ethics committee of the Thailand Institute for the Development of Human Subject Protection (reference IHRP2021059) and the Oxford University Tropical Research Ethics Committee (OxTREC 529-21).

We will adhere to the relevant guidelines for qualitative research. All interviewers, and transcribers will be trained prior to the study. The project and its evaluation will be conducted in accordance with relevant Thai and international guidance and regulations. The protocol, informed consent form, participant information sheet and any associated material will be submitted to relevant ethics committees for written approval.

### Risk and benefits

This is a minimal risk and minimal harm project. The main concerns relate to privacy and confidentiality. We will make every effort to maintain privacy of participants during the audio recording of the conversations as well as evaluation interviews and FGDs. The participants will be identified only by a participant reference number on all project documents and databases. All documents will be stored securely and only accessible by authorised study personnel. We will comply with the UK General Data Protection Regulation (GDPR) and Thailand Personal Data Protection Act (PDPA).

The other concern revolves around COVID-19 risks. We will comply with regional and national COVID-19 guidelines. Where in-person conversations or focus group discussions (for evaluation) are planned, but are not possible due to COVID-19, we will convert them to virtual sessions.

There are no anticipated direct benefits to the participants apart from learning about AMR and having their views heard. New knowledge gained from this initiative is expected to inform Thailand’s National Strategic Plan on Antimicrobial Resistance committee for improving policies to reduce the burden of AMR across Thailand. Participant will receive the compensation for their time to participate in the conversations (1000 Thai baht/GBP 25 per session) and evaluation focus group discussion and in-depth interviews (300 Thai baht/GBP7.50 per session).

### Public involvement

This protocol describes a public involvement project. As part of the development of our study and data collection tools, we have conducted a series of meetings with the Bangkok Health Research Ethics Interest Group (BHREIG) ^32^, including the activities in the online national conversation with BHREIG.

### Dissemination of information

The learning and outcomes will be disseminated to policy makers, organisers of the Thailand AMR week, Wellcome Trust, academic audiences, as well as back to participants of all the conversation events. Some of the avenues include:

•    Talks presented at conferences/meetings targeting engagement practitioners who do AMR work

•    Blogs on the MESH website targeting engagement practitioners and other relevant websites

•    Public talks and panel discussions conducted through MORU’s and its partners’ existing engagement channels e.g. BHREIG, science cafes, and Pint of Science events ^33– 36^.

•    Dialogues with professional groups e.g. pharmacists in Thailand and beyond, focusing on the co-created AMR solutions. 

•    The final AMR stakeholder map, an interim project report and final project report of the project will be provided to the Thailand’s National Strategic Plan on Antimicrobial Resistance committee.

### Strengths and limitations 

Strengths: This is the first time Thailand’s National Strategic Plan on Antimicrobial Resistance will have direct input from members of the public and other stakeholders previously not consulted. It is envisaged that embedding voices from the public will make the AMR policy more context-specific and realistic.

Limitation: We plan to invite participants from minority groups, from different age groups, social groups, and educational levels to attend the conversations. We recognize there may be power dynamics and other such challenges that may hinder some participants from making their voices heard. 

There may be also challenges due to the team’s positionality as some of the members have been involved in current and previous Thailand’s National Strategic Plans on Antimicrobial Resistance which may deter some participants from giving us honest opinions during the conversations. 

In facilitating the conversations, we will pay extra attention to group dynamics and encourage those who are less vocal to speak. Our facilitation team members are experienced in conducting workshops with members of the public and communities with a wide range of backgrounds. We also plan to have breakout groups as some participants may feel more comfortable speaking up in a smaller group. 

### Study status

Protocol approvals have been obtained and conversations have commenced.

## Data availability

### Underlying data

No data are associated with this article.

### Extended data

Zenodo: Extended data for "AMR Dialogues": a public engagement initiative to shape policies and solutions on antimicrobial resistance (AMR) in Thailand. http://doi.org/10.5281/zenodo.5115834 ^29^.

This project contains the following extended data:

- AMR Dialogues evaluation PISICF_V3.0 21Jun2021.docx (participant information sheet, consent form)
- AMR Dialogues evaluation_Question guides_v3.0 21Jun2021.docx

Data are available under the terms of the Creative Commons Attribution 4.0 International license (CC-BY 4.0).
