# Supplementary material for: “AMR Dialogues”: a public engagement initiative to shape policies and solutions on antimicrobial resistance (AMR) in Thailand
Source: Wellcome Open Res. 2022 Apr 7;6:188. Originally published 2021 Jul 26. [Version 2] doi: 10.12688/wellcomeopenres.17066.2 (PMC9034167; doi:10.12688/wellcomeopenres.17066.2)
Supplement: Peer review history

# Peer review: Reviewer response for version 2

**DOI:** 10.21956/wellcomeopenres.19733.r49812

**Published:** 2022-04-22

**Authors:** Papreen Nahar

**Affiliations:** 1 Department of Global Health and Infection, Brighton and Sussex Medical School, Sussex University, Brighton, UK

I have no further comments to make.

Is the study design appropriate for the research question?

Yes

Is the rationale for, and objectives of, the study clearly described?

Yes

Are sufficient details of the methods provided to allow replication by others?

Partly

Are the datasets clearly presented in a useable and accessible format?

Yes

Reviewer Expertise:

Antimicrobial Resistance, Infertility, Neglected Tropical Diseases, Non-communicable disease

I confirm that I have read this submission and believe that I have an appropriate level of expertise to confirm that it is of an acceptable scientific standard.

# Peer review: Reviewer response for version 1

**DOI:** 10.21956/wellcomeopenres.18852.r46589

**Published:** 2021-12-08

**Authors:** Papreen Nahar

**Affiliations:** 1 Department of Global Health and Infection, Brighton and Sussex Medical School, Sussex University, Brighton, UK

This protocol paper presents the public engagement initiative on AMR in Thailand. AMR is a global public health threat that requires a local solution. This protocol provides a timely and appropriate example of such a local initiative. Public engagement is the core element of the co-creation of public health interventions and this protocol provides an organized and very systematic approach for public engagement in Thailand to find out context-specific solutions. The protocol takes a ‘dialogue’ approach to engage the community and the researchers also propose to involve a range of very relevant stakeholders who can play a role in mitigating ARM in Thailand. The sequence of the engagement is also appropriate, which begins with exploring the issue and then brainstorming for solutions. It is good that the protocol includes an evaluation element as well. It is also appreciable that the team considered both in-person and virtual public engagement events keeping the pandemic situation in mind.

 However, I have the following queries and clarifications:

- It is good that the research team has suggested several diverse groups with whom they intend to have the conversation. However, the group's compositions seem too generalized in some cases. Although the groups have been categorised through different attributes, there might be heterogenicity within the group. For example, there could be huge diversity between users of antibiotics for humans or animals in terms of economic status, education, gender. Among the ‘solution experts’, there might be a difference in terms of status and experience. All these differences might influence the conversation if done as a group. I would recommend paying attention to this particular aspect of group dynamics.
- The protocol proposes seven community conversation, however, eleven categories of community has been listed. Not clear how this will be resolved.
- Informal providers are major health care providers, who dispense and prescribes antibiotics in most of the global south countries. Although the protocol acknowledges their role in Thailand, the team does not include them in the conversation. I think this needs attention.
- Some clarification is needed regarding the evaluation. It has been proposed that an evaluation of the public engagement will be done in two stages one right after the conversation and the other after one month on the following issues 1) their understanding or increased knowledge on AMR, 2) their level of awareness of AMR, and 3) their actions or solutions they think they would and could do regarding AMR. It is understandable that the response just after the dialogue will be influenced by the close time immediacy of the dialogue. It is not clear whether the same set of questions will be used during evaluation after one month. Also, it is not clear whether the individual impact of the dialogue could be evaluated in Focus Group Discussion. 

Is the study design appropriate for the research question?

Yes

Is the rationale for, and objectives of, the study clearly described?

Yes

Are sufficient details of the methods provided to allow replication by others?

Partly

Are the datasets clearly presented in a useable and accessible format?

Yes

Reviewer Expertise:

Antimicrobial Resistance, Infertility, Neglected Tropical Diseases, Non-communicable disease

I confirm that I have read this submission and believe that I have an appropriate level of expertise to confirm that it is of an acceptable scientific standard, however I have significant reservations, as outlined above.

# Response

**Published:** 2022-04-03

**Authors:** Phaik Yeong Cheah

**Affiliations:** Mahidol Oxford Tropical Medicine Research Unit, Thailand

Many thanks for your valued comments. Here is our point by point response. 

 This protocol paper presents the public engagement initiative on AMR in Thailand. AMR is a global public health threat that requires a local solution. This protocol provides a timely and appropriate example of such a local initiative. Public engagement is the core element of the co-creation of public health interventions and this protocol provides an organized and very systematic approach for public engagement in Thailand to find out context-specific solutions. The protocol takes a ‘dialogue’ approach to engage the community and the researchers also propose to involve a range of very relevant stakeholders who can play a role in mitigating ARM in Thailand. The sequence of the engagement is also appropriate, which begins with exploring the issue and then brainstorming for solutions. It is good that the protocol includes an evaluation element as well. It is also appreciable that the team considered both in-person and virtual public engagement events keeping the pandemic situation in mind.

 However, I have the following queries and clarifications:

 It is good that the research team has suggested several diverse groups with whom they intend to have the conversation. However, the group's compositions seem too generalized in some cases. Although the groups have been categorised through different attributes, there might be heterogenicity within the group. For example, there could be huge diversity between users of antibiotics for humans or animals in terms of economic status, education, gender. Among the ‘solution experts’, there might be a difference in terms of status and experience. All these differences might influence the conversation if done as a group. I would recommend paying attention to this particular aspect of group dynamics.

**Response:** We agree with your comments.  We recognize that this is a potential limitation to get all voices heard. We have included a new section “Limitations” to acknowledge this explained that we will pay attention to group dynamics as well as power dynamics.

 The protocol proposes seven community conversation, however, eleven categories of community has been listed. Not clear how this will be resolved.

**Response:** Most of the conversations will have a mixture of groups from these 11 categories (now 12 – see next response)

 Informal providers are major health care providers, who dispense and prescribes antibiotics in most of the global south countries. Although the protocol acknowledges their role in Thailand, the team does not include them in the conversation. I think this needs attention.

**Response:** We agree. We have now included this group in “Participant selection criteria for community conversations”.

 Some clarification is needed regarding the evaluation. It has been proposed that an evaluation of the public engagement will be done in two stages one right after the conversation and the other after one month on the following issues 1) their understanding or increased knowledge on AMR, 2) their level of awareness of AMR, and 3) their actions or solutions they think they would and could do regarding AMR. It is understandable that the response just after the dialogue will be influenced by the close time immediacy of the dialogue. It is not clear whether the same set of questions will be used during evaluation after one month.

**Response:** The questions are not the same. We have updated paragraph 7 in Part 2. Please see question guide here https://zenodo.org/record/5115834#.YkMSuzfMLeo. We have included this in the reference.

 Also, it is not clear whether the individual impact of the dialogue could be evaluated in Focus Group Discussion. 

**Response:** Thank you for the comment. It is true that individual impact cannot be evaluated in focus group discussions. We will include individual interviews as well.

 Thank you for all your comments.

# Peer review: Reviewer response for version 1

**DOI:** 10.21956/wellcomeopenres.18852.r46247

**Published:** 2021-10-12

**Authors:** David Kaawa-Mafigiri

**Affiliations:** 1 Center for Social Sciences Research on AIDS, School of Social Sciences, Makerere University College of Humanities and Social Sciences, Kampala, Uganda; 2 Department of Anthropology, Case Western Reserve University, Cleveland, OH, USA

This protocol seeks to utilise an community engagement model (Dialogues with stakeholders) to influence policy making for controlling AMR in Thailand. It adopts a 'bottom-up' approach to ensure that stakeholders, particularly community members exposed to the problem participate in developing policy towards their wellbeing. It is an important contribution to the evidence on how to engage communities in solving their problems and improving their wellbeing. Specifically, for contemporary social problems like the growing epidemics of AMR, infectious disease outbreaks and other humanitarian emergencies, it is now clear that the role of non-biomedical interventions is as important in combating them, as has been the case with biomedical technology. Therefore, the authors propose a much-needed protocol to try and understand how concerned communities can be engaged and participate in shaping policies and solutions for antimicrobial resistance in Thailand.

 The background, including literature on the problem of AMR in Thailand is well stated. Perhaps there may be need to expound a little more on how antibiotics and anti-inflammatory drugs are perceived or conflated in the local context. What examples of language illustrate this conflation? Expounding, with one example will enhance the background.

 It was also not clear what the following statement means: “In Thailand, by law, most antimicrobials can be dispensed by licensed pharmacists at pharmacies without a prescription.” - Do you imply that it is not illegal to dispense antimicrobials without prescription? Or that the law is not followed concerning dispensing antimicrobials but when the law could be that they shouldn’t be dispensed without a prescription? It may be helpful to cite the law in this case.

 Objective 2 is quite important as there is a need to provide a catalyst to AMR policy while recognizing that change or improvement should be context specific and locally-driven. As such I would think that rather than ‘drive change’ the team wishes to ‘provide a catalyst for improving current AMR policy’.

 The protocol is well planned. However, there are a series of activities being planned which may require a bit more elaboration on how they are tied to the or would contribute to ‘catalyzing’ change in policy. Perhaps a matrix or chart may help to tag or identify what planned meetings or sessions lie under the respective policy areas being targeted. For instance, given that there are different levels of actors whose contribution/participation is being sought, then perhaps stratifying the meetings or sessions by target populations would help the reader chart the flow of how these meetings may enhance or contribute to policy change. Similarly there were several formats of the meetings that need some clarification (e.g. would virtual breakout sessions be part of the 20-25 in-person meetings?). That is an example where it is not clear if the virtual breakout session is part of an in-person meeting such that as the in-person meeting is ongoing, there is an opportunity for the same teams to break out into sub groups but virtually?

 Part 1: AMR Dialogues: Should all conversations be led by the research team? Is it possible to consider if some of the communities of participants being involved can lead some conversations – in a systematic way – without compromising on the design? Since the protocol is premised on the concept of community engagement – should the engagement entail the community leading in some of the sessions?

 The study proposes sessions of 20-25 persons. Given the large numbers in the context of COVID-19, it would be good to have a note on what risk mitigation measures are in place or how those would influence/impact the method of conversation. See also the suggestion to have up to 12 participants in an FGD. What are the implications if there was COVID-19 restrictions in Thailand and how would it influence the protocol? A short note on the situation of the pandemic and how it is affecting research may help clear this. It could be elaborated under the section “risks and benefits”.

 Similarly, the protocol indicates that attendees of all gender are welcome. Will age be a variable to consider and if so will it be varied or grouped and by what criteria? If not, a note on that is helpful for the reader to determine the justification or possible limitation. This comment is inspired by knowing that in some societies age determines whose opinion counts during conversations/meetings or planning for social issues.

 The team plans to invite diverse stakeholders through their professional networks of previous engagement in AMR related or other health projects. Would the team comment about the issue of positionality and whether it is a potential limitation to the study?

 The sample size estimation/description is not clearly stated. Are the approximately 70-85 participants for FGDs only? It may be best to simply state the number of FGDs estimated, then separately assign the number of In-depth interviews planned.

 Mandatory questions:

1. Is the rationale for, and objectives of the study clearly stated?
  Partly. See further comments above under background. Objective 2 is quite important as there is a need to provide a catalyst to AMR policy while recognizing that change or improvement should be context specific and locally-driven. As such I would think that rather than ‘drive change’ the team wishes to ‘provide a catalyst for improving current AMR policy’.
2. Is the study design appropriate for the research question?
  Yes
3. Are sufficient details of the methods provided to allow replication by others?
  Yes
4. Are the datasets clearly presented in a usable and accessible format?
  Yes

Is the study design appropriate for the research question?

Yes

Is the rationale for, and objectives of, the study clearly described?

Partly

Are sufficient details of the methods provided to allow replication by others?

Yes

Are the datasets clearly presented in a useable and accessible format?

Yes

Reviewer Expertise:

Medical Anthropology, infectious disease control, community based interventions

I confirm that I have read this submission and believe that I have an appropriate level of expertise to confirm that it is of an acceptable scientific standard.

# Response

**Published:** 2022-04-03

**Authors:** Phaik Yeong Cheah

**Affiliations:** Mahidol Oxford Tropical Medicine Research Unit, Thailand

Many thanks for your valued comments. We appreciate it very much. Here is our point by point response.

 This protocol seeks to utilise an community engagement model (Dialogues with stakeholders) to influence policy making for controlling AMR in Thailand. It adopts a 'bottom-up' approach to ensure that stakeholders, particularly community members exposed to the problem participate in developing policy towards their wellbeing. It is an important contribution to the evidence on how to engage communities in solving their problems and improving their wellbeing. Specifically, for contemporary social problems like the growing epidemics of AMR, infectious disease outbreaks and other humanitarian emergencies, it is now clear that the role of non-biomedical interventions is as important in combating them, as has been the case with biomedical technology. Therefore, the authors propose a much-needed protocol to try and understand how concerned communities can be engaged and participate in shaping policies and solutions for antimicrobial resistance in Thailand.

**Response:** Thank you.

 The background, including literature on the problem of AMR in Thailand is well stated. Perhaps there may be need to expound a little more on how antibiotics and anti-inflammatory drugs are perceived or conflated in the local context. What examples of language illustrate this conflation? Expounding, with one example will enhance the background.

**Response:** We have now included a sentence in paragraph 3.

 It was also not clear what the following statement means: “In Thailand, by law, most antimicrobials can be dispensed by licensed pharmacists at pharmacies without a prescription.” - Do you imply that it is not illegal to dispense antimicrobials without prescription? Or that the law is not followed concerning dispensing antimicrobials but when the law could be that they shouldn’t be dispensed without a prescription? It may be helpful to cite the law in this case.

**Response:** We have now cited the law. See paragraph 2.

 Objective 2 is quite important as there is a need to provide a catalyst to AMR policy while recognizing that change or improvement should be context specific and locally-driven. As such I would think that rather than ‘drive change’ the team wishes to ‘provide a catalyst for improving current AMR policy’.

**Response:** We have now changed the text as advised.

 The protocol is well planned. However, there are a series of activities being planned which may require a bit more elaboration on how they are tied to the or would contribute to ‘catalyzing’ change in policy. Perhaps a matrix or chart may help to tag or identify what planned meetings or sessions lie under the respective policy areas being targeted. For instance, given that there are different levels of actors whose contribution/participation is being sought, then perhaps stratifying the meetings or sessions by target populations would help the reader chart the flow of how these meetings may enhance or contribute to policy change.

**Response: **Our project is specifically aimed at only one strategy of the National Action Plan. All the activities are targeting this strategy which is Strategy 5 (public knowledge and awareness of appropriate use of antimicrobials). We have made it clearer in the text. See objective 2.

 Similarly there were several formats of the meetings that need some clarification (e.g. would virtual breakout sessions be part of the 20-25 in-person meetings?). That is an example where it is not clear if the virtual breakout session is part of an in-person meeting such that as the in-person meeting is ongoing, there is an opportunity for the same teams to break out into sub groups but virtually?

**Response:** That was an error. Where there are in-person meetings, the breakout rooms will be in-person. Similarly, where there are virtual meetings, all breakout rooms will be virtual. We have clarified this. See Phase II. Community Conversations.

 Part 1: AMR Dialogues: Should all conversations be led by the research team? Is it possible to consider if some of the communities of participants being involved can lead some conversations – in a systematic way – without compromising on the design? Since the protocol is premised on the concept of community engagement – should the engagement entail the community leading in some of the sessions?

**Response:** Thank you for the suggestion. The Wellcome Responsive Dialogues toolkit is very specific and the project team are following the guide closely. Hence, we feel that we should not ask community participants who have no training using Wellcome’s toolkit to lead the main sessions. We have also described the project team’s background. We have vast experience in engagement and facilitation. However, we think it is a good idea to involve community members to lead the discussions. We will ask some of them to lead the breakout group discussions. See Part 1 paragraph 2.

 The study proposes sessions of 20-25 persons. Given the large numbers in the context of COVID-19, it would be good to have a note on what risk mitigation measures are in place or how those would influence/impact the method of conversation. See also the suggestion to have up to 12 participants in an FGD. What are the implications if there was COVID-19 restrictions in Thailand and how would it influence the protocol? A short note on the situation of the pandemic and how it is affecting research may help clear this. It could be elaborated under the section “risks and benefits”.

**Response:** Thank you for the suggestion. We have now included a paragraph on COVID-19 mitigation plans.

 Similarly, the protocol indicates that attendees of all genders are welcome. Will age be a variable to consider and if so will it be varied or grouped and by what criteria? If not, a note on that is helpful for the reader to determine the justification or possible limitation. This comment is inspired by knowing that in some societies age determines whose opinion counts during conversations/meetings or planning for social issues.

**Response:** This is indeed a good point. We intend to invite all age groups but we recognize that there may be challenges in mixing age groups. In Thailand, opinions of older people are respected. See paragraph 3 in Part 1, and the new section on Strengths and Limitations.

 The team plans to invite diverse stakeholders through their professional networks of previous engagement in AMR related or other health projects. Would the team comment about the issue of positionality and whether it is a potential limitation to the study?

**Response:** Thank you. We have added a section on Limitation to address this point.

 The sample size estimation/description is not clearly stated. Are the approximately 70-85 participants for FGDs only? It may be best to simply state the number of FGDs estimated, then separately assign the number of In-depth interviews planned.

**Response:** We expect to run four FGDs of between 6–12 participants per FGD. See Evaluation (paragraph 4). We have included the number (70-85) at the request of our ethics committee.

 We have also updated some text for clarity. Please also see our response to the other reviewer. 

 Thank you,.
